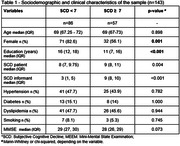# Should the perspective of subjective memory decline differ between cognitively unimpaired individuals and their informants? Baseline results of the BRASCODE Cohort

**DOI:** 10.1002/alz.093103

**Published:** 2025-01-09

**Authors:** Rhaná Carolina Santos, Sarah Vitoria Bristot Carnevalli, Haniel Bispo De Souza, Victória Tizeli Souza, Manuella Edler Zandoná Giordani, Simone Sieben da Mota, Bruno De Olive De Marchi, Carolina Rodrigues Formoso, Gabriela Raquel Paz Rivas, Guilherme Da Silva Carvalho, Lucas Bastos Beltrami, Samuel Masao Suwa, Wesley Slaviero, Ana Letícia Amorim de Albuquerque, Matheus Zschornack Strelow, Wyllians Vendramini Borelli, Giovanna Carello‐Collar, Marcia L Fagundes Chaves, Eduardo R. Zimmer, Raphael Machado Castilhos

**Affiliations:** ^1^ Universidade do Vale do Rio dos Sinos, São Leopoldo, Rio Grande do Sul Brazil; ^2^ Universidade Federal de Santa Catarina, Florianópolis Brazil; ^3^ Universidade Federal do Rio Grande do Sul, Porto Alegre, RS Brazil; ^4^ Hospital de Clínicas de Porto Alegre, Porto Alegre, Rio Grande do Sul Brazil; ^5^ Universidade Federal do Rio Grande do Sul, Porto Alegre Brazil; ^6^ Federal University of Rio Grande do Sul, Porto Alegre, Rio Grande do Sul Brazil; ^7^ Memory Center, Hospital Moinhos de Vento, Porto Alegre, RS Brazil; ^8^ Clinical Hospital of Porto Alegre, Porto Alegre, Rio Grande do Sul Brazil; ^9^ Universidade Federal do Rio Grande do Sul, Porto Alegre, Rio Grande do Sul Brazil; ^10^ Brain Institute of Rio Grande do Sul (InsCer), PUCRS, Porto Alegre, Rio Grande do Sul Brazil; ^11^ Federal University of Rio Grande do Sul (UFRGS), Porto Alegre, RS Brazil; ^12^ Brain Institute of Rio Grande Do Sul, PUCRS, Porto Alegre, RS Brazil; ^13^ McGill Centre for Studies in Aging, Montreal, QC Canada; ^14^ Memory Center, Hospital Moinhos de Vento, Porto Alegre, RS, Brazil Brazil

## Abstract

**Background:**

Subjective Cognitive Decline (SCD) is characterized by cognitive complaints in cognitively unimpaired (CU) individuals. Its condition displays considerable heterogeneity etiologies, including neurodegenerative diseases. Our aim is to compare the memory complaints between patients with SCD and their informants in the BRASCODE cohort.

**Method:**

The BRASCODE cohort includes CU individuals aged >65 presenting memory complaints and at least one SCD‐plus criteria. The MCS was held separately with both participants and informants. Additionally, baseline sociodemographic and Mini Mental State Examination (MMSE) data were held. Participants and informant’s concordance regarding patients' memory complaints was assessed using the MCS scale. If both answers were ≥ 7, there was concordance. The continuous variables were described as median (interquartile range, IQR) and categorical variables as frequencies. Scores of the MCS scale ranges from 0 to 14, with scores ≥ 7 indicating complaint; and were compared using Spearman’s rho and Mann‐Whitney test.

**Result:**

Data were collected between March‐2022 and December‐2023. 143 SCD patients were included (mean age = 69; 72.2% female). The median MCS‐patient and MCS‐informant was 8 and 5, respectively. The patients have a median of 3 SCD‐plus criteria, most with complaints at age ≥ 60 and with concerns regarding memory. The SCD‐patient scale correlated negatively with patient age (rho = ‐0.244; p = 0.003) and positively with informant’s SCD scale (rho = 0.305; p = 0.0002), but not with patient education nor with MMSE results. Most participants (86, 60.1%) and their informants' perceptions disagree about patients' memory decline. The participants in the group with concordant MCS scores had lower education (11 vs 16) and were more balanced in relation to sex (32 [56.1], 71 [82.6]) than the group with discordante responses. The patients' age and MCS were not different between the groups.

**Conclusion:**

The baseline results show that most dyads disagreed regarding participants' memory decline. In addition, the presence of informant perception of decline may be a risk factor for a future cognitive decline. The AD biomarkers analysis as well as one and two‐year follow‐ups may yield valuable insights into the correlation between patients' and informants' reported complaints.